# Temporary Threshold Shifts among Iron and Steel Factory Workers in Tanzania: A Pre-Interventional Study

**DOI:** 10.5334/aogh.3193

**Published:** 2021-04-06

**Authors:** Israel P. Nyarubeli, Magne Bråtveit, Alexander Mtemi Tungu, Simon H. Mamuya, Bente E. Moen

**Affiliations:** 1 Department of Environmental and Occupational Health, Muhimbili University of Health and Allied Sciences, P.O. Box 65015, Dar es Salaam, Tanzania; 2 Research Group for Occupational and Environmental Medicine, Department of Global Public and Primary Care, University of Bergen, 5020 Bergen, Norway; 3 Department of Physiology, Muhimbili University of Health and Allied Sciences, P.O. Box 65001, Dar es Salaam, Tanzania; 4 Centre for International Health, Department of Global Public and Primary Care, University of Bergen, Årstadveien 21, 5020 Bergen, Norway

## Abstract

**Background:**

Workers in iron and steel factories in Tanzania are exposed to noise levels above recommended limit values, without using hearing protection devices. Exposure to noise levels above 85 dB(A) is associated with temporary threshold shifts (TTS) of human hearing. Nevertheless, there are few studies of noise and hearing from African countries.

**Objective:**

To determine whether the normal hearing workers in Tanzania experiences TTS after full-shift occupational noise exposure of 85 dB(A) and above.

**Methods:**

A total of 55 workers were included. Full-shift personal noise measurements were conducted. Pre- and post-shifts pure-tone audiometry were conducted for each worker. TTS was defined as a 10 dB or greater change at 1000, 2000, 3000 or 4000 Hz in either ear.

**Results:**

We found that 85.5% of the workers developed TTS across the work shift. There was significant increase in mean hearing thresholds across shift at 1000, 2000, 3000 and 4000 Hz among the workers exposed to an average personal noise exposure (L_Aeq,8h_) of 90.4 dB(A) (SD = 2.7). The difference in mean hearing thresholds was higher at 4000 Hz [Arithmetic Mean (AM) = 10 dB SD = 4 dB] compared to that of 1000 Hz (AM = 4 dB SD = 3 dB), 2000 Hz (AM = 4 dB SD = 4 dB), and 3000 Hz (AM = 9 dB SD = 6 dB), respectively.

**Conclusions:**

Interventions to reduce occupational personal noise exposure are warranted to reduce the high risk of developing a permanent threshold shift with persistent high noise exposure. An intervention study is planned for this group of workers.

## Introduction

Noise-induced hearing loss (NIHL) is a slowly progressive, sensorineural hearing decrement, which typically occurs at higher frequencies (3–6 kHz) as a result of prolonged exposure to high intensity sound levels [1]. It is one of the most commonly reported occupational-related problems in industrial workplaces [2,3]. It is estimated that 466 million people live with disabling hearing loss globally, one-third of which is attributed, at least in part, to noise exposure [4,5]. Industries affected by this problem are, for instance, manufacturing industries in different parts of the world, including Sub-Saharan Africa (SSA) [2]. Identifying workers-at-risk offers a great opportunity for targeted interventions that may benefit workers from developing NIHL [6], and ultimately society.

High noise levels and increased NIHL prevalence have been reported for workers in many industries in Tanzania, for instance among textile production workers (≥90 dB with 40% hearing loss) [7], workers in small scale metal industrial areas (87–117 dB[A]) [8], workers in gas-fired electric plants (L_Aeq_,_8hr_ of 98 SD = 9.7 with 53.8% subjective hearing loss) [9], and among cement factory workers (L_Aeq_ of 70–104 dB[A]) where the subjective hearing loss was 54% [10]. In addition, a recent study among iron and steel workers also found high noise exposure levels (*L*_Aeq,8h_ = 92 dB[A]) [11], and a high prevalence of NIHL (48%) [12,13].

Temporary threshold shift (TTS), which is the reversible hearing loss that occurs immediately after exposure to intense sound levels, and which recovers over a period of several hours or days after exposure, is considered an important indicator for the early development of irreversible hearing loss [14]. Thus, monitoring TTS after noise exposure among workers is an alternative and promising approach to facilitate the prevention of hearing loss [15]. To quantify TTS, several methods and definitions have been used including different combinations of frequencies and threshold shifts. Recent studies in human subjects have used the definition of a shift of 10 dB or greater change at 1, 2, 3 or 4 kHz in either ear between two consecutive audiometric measurements [16], for instance between pre- and post-shift, among noise exposed workers.

Few field studies have been conducted in humans on TTS and none among the iron and steel workers in Tanzania. These studies show increased hearing thresholds, and a high prevalence of TTS among noise-exposed workers. For example, a study in the United States involving male, hockey officials found a TTS of 86.2% at an equivalent noise exposure of 93 dB(A) ranging from 88–97 dB(A) [17]. A TTS prevalence of 77% was reported among bartenders in Poland exposed to an average noise level of 95 dB(A) [18]. Similarly, an experimental study showed an increase in hearing threshold of 6.3 (SD = 3.9) among digital music players exposed to noise levels of 93–102 dB(A) for a duration of four hours [19]. All together, these studies suggest a relationship between high noise exposure and the risk of TTS.

Although the Tanzanian Occupational Safety and Health Act (OSHA) requires the employer to provide and maintain effective personal protective equipment (including Hearing Protection Devices [HPDs]) for use by employees who are exposed to hazardous levels of noise [20], a recent study found that no worker in the iron and steel factories in Tanzania used HPDs [11]. It seems vital to introduce HPDs as a first intervention step to protect these workers from the adverse effect of noise on hearing.

In this study, we assumed that the iron and steel workers exposed to 92 dB(A)would develop TTS [13], and that this could have been prevented by the proper use of HPD, following instructions on how to use the devices correctly. However, the protective effect of this intervention is dependent on the actual noise attenuation and the sustainability of HPD use among the workers. To our knowledge, no such published data are available from Tanzania or any other Sub-Saharan African countries. Furthermore, despite the knowledge about the long-term effect of prolonged exposure to hazardous noise on hearing, the impact of daily (full shift) personal noise exposure of hearing sensitivity (threshold) is poorly documented. An intervention study was therefore planned to be the starting point for a hearing conservation programme where workers will be provided with HPDs. TTS will be used as an indirect effect measure of the noise attenuation. The advantages of using TTS as an indicator is that the measurements require short time for completion, are not too costly and have low potential for participants attrition [19]. This study presents the results of an initial, pre-intervention assessment of TTS among iron and steel factory workers in Tanzania. The purpose was to determine whether or not workers with normal hearing, experience TTS during full-shift occupational noise exposures of 85 dB(A) and above. This pre- intervention assessment was undertaken a week before implementation of the full intervention, and therefore resolving the ethical dilemma of involving people at work in this project [14,21].

## Materials and Methods

### Study population

This study involved male production workers in one iron and steel factory in Dar es Salaam, Tanzania. No women were working on the production line. The factory has a total of 200 workers on the production line and its production capacity is about 20,000 tons of rebars per year. The factory is among four iron and steel factories in Tanzania that have been previously studied for workers’ exposure to noise in the production area and their respective NIHL [12,13]. The workers did not use HPDs while working in the noisy environment [11].

### Study participants

The factory administration allowed us to perform the study at their site. From a recent list of workers provided by the factory administration in 2020 together with the information available from the previous study, i.e. job titles, work sections, shift pattern, description of tasks and work environment [12]. We invited workers from the production area in the factory with noise levels above 85 dB(A) [22], i.e. working in rolling mills and furnace sections, to participate in our study. We explained the purpose and activities of the study and asked for the informed consent of each participating worker. Initially, the required sample size for this study was estimated to be 47 workers [23]. To be selected into this study, the inclusion criteria were those workers with otologically normal hearing, i.e., workers with air conduction average hearing thresholds (measured through Pure Tone Audiometry [PTA]) better than 25 dB for the sound frequencies 0.25–8 kHz and having no signs of any other ear disease [19,24]. In this process, we screened 66 participants out of which 10 were ineligible, as six had hearing loss (HTL > 25 dB), two had a perforated tympanic membrane and two had ear infections. In addition, one worker declined to participate. Thus, the study recruited 55 eligible workers. We collected baseline characteristics for the study participants by a short interview asking for their age (years), duration of work (years), educational level, current smoking (yes/no), use of earphones/headphones for listening to music (yes/no), being on long term medication (yes/no) and living in areas with high noise sources such as industry, high music, workshop (yes/no).

### Personal noise measurement

We conducted full-shift personal noise measurements using personal noise dosimeters (Brüel and Kjaer type 4448, DK-2850 Nærum, Denimark) following the ISO standard 9612:2009 [25]. The dosimeters had a measurement range from 50–140 dB (A – weighted noise level [*L_p_*_,A_]), and were set up at a 3-dB exchange rate. The dosimeters logged noise data each minute during the measurement period. We calibrated the dosimeters before and after each sampling period. During the measurement period, we attached the dosimeters to the workers’ shoulders approximately 10–15 cm from the ear and instructed the participants to handle the dosimeters carefully while working, by not touching, tampering, or shouting into the dosimeter’s microphone. In addition, we conducted several follow-ups during the measurement period to check for any dosimeter mishaps. After each measurement day, we recorded the start- and end-time of the sampling period, the A-weighted equivalent noise level for the duration of the measurement (*L_p_*_,A,eq_*_T_*) and the C-weighted peak noise level (*L_p_*_,Cpeak_). The data were normalized to daily noise exposure levels (*L*_EX,8h_) by noise exposure groups (job groups) and working sections, using the following formula:

{L_{{\rm{EX,8h}}}} = {{\rm{L}}_{p{\rm{,A,eq}}T{\rm{e}}}} + {\rm{10}}{\log ^{(T{\rm{e}}/T0)}}

Where; *L_p_*_,A,eq_*_T_*_e_ is the A-weighted equivalent continuous sound pressure level from dosimeter, *T*e is the measurement period and *T*0, is the reference duration, equal to eight hours. This was done to see if there was any variability in personal noise exposure with the previous personal noise exposure profile [13].

### Audiometry

The PTA for each study participant was conducted in a mobile soundproof booth between February and March 2020 along with other personal noise measurements. The same technical personnel conducted all audiometric tests using a standardized protocol. Background noise in the test booth was monitored by a calibrated hand-held Sound Level Meter (Brüel and Kjær, type 2250), and the test environment conformed with the requirement of ISO 8253–1:2010 standard [26]. Pre-shift PTA were measured in the morning before workers started their working shift and post-shift PTA were conducted immediately after the workers finished the daily working-shift. The day shift started at 07:00 a.m. in the morning and ended at 17.00 p.m. in the evening. Measurements were assumed to be consistent at both times of measurement as any diurnal variability in hearing threshold is not likely to occur [27,28]. PTA was conducted using an Interacoustics AD226 (Interacoustics, DK-5500 Middelfart, Denimark) with Amplivox Audiocup earphones having lower test limit of –10 dB. The equipment was pre-calibrated. Test frequencies were 250 – 8000 Hz in the order starting with 1000, 2000, 3000, 4000, 6000, 8000, 500, 250 and finishing at 1000 Hz [26]. A manual test procedure was used in compliance with ISO 8253–1:2010 [26,29,30]. Temporary threshold shift (TTS) was defined as a 10 dB or greater change in hearing threshold between pre-shift and post-shift.at 1000, 2000, 3000 or 4000 Hz in either ear [16].

### Ethical clearance

We obtained ethical clearance from two ethical boards namely, The Regional Committee of Medical and Health Research Ethics (REK-VEST) in Norway and from The Muhimbili University of Health and Allied Sciences (MUHAS) Ethics Committee in Tanzania. We held meeting with the factory administration where we presented the purpose of the project and asked for a project permit. We were provided with a list of existing 200 workers. Workers were informed of the purpose of the project and those who agreed to participate, provided written consent. The information collected was treated with confidentiality and was not accessible to anyone other than the researchers involved in examining the workers.

### Data analysis

We present descriptive statistics as mean and standard deviation or percentage.

Paired samples t-test were used to compare the participant’s mean hearing thresholds between pre- and post- shifts audiometry at 1000, 2000, 3000, and 4000 Hz, respectively. An average personal noise exposure (*L*_Aeq,8h_) was consolidated and computed using Microsoft Excel (from MS office 365).

An overall TTS (yes, no) was computed. Potential determinants for hearing threshold shifts including age and duration of work variables were categorized into three groups (tertiles), while other variables were dichotomized; Current smoking (yes/no), being on long term medication (yes/no) and the use of earphones/headphones for listening to music/radio at work or home (yes/no). No one had experienced any activities and or machines that generated noise at or just close to their home, and only one participant reported being on long term medication. This entry was left out in the analysis because the participant did not remember the type of medicine nor the duration for which he had taken the medication.

Correlations between participant’s age and duration of work as well as between noise peaks and average personal noise exposure were tested using the Pearson correlation test.

The IBM SPSS Statistics for Windows, version 25 (IBM corp., Armok, N.Y., USA) was used for the data analysis and a parameter of p < 0.05 was set as indicating statistical significance.

## Results

The study participants’ mean age was 31 (SD = 7; range: 20–47) years. The mean work-duration was 6 (SD = 5; range: 1–20) years. Workers were exposed to an average personal noise (*L*_Eq,8h_) of 90.4 (SD = 2.7, ranging from 85.0–95.5 dB [A]). The average C-weighted peak noise level (Lp, Cpeak) was 134.1 (SD = 6.1; range 107.8–143.5) dB(C). The mean number of noise peaks >130 dBC was 12 dB(C) peaks per personal noise measurement (SD = 9.7, ranging from 1–52) dB(C). Thirteen percent of the workers were current smokers and the same percentage used earphones/headphones for listening to music/radio at work or at home (***Table 1***).

**Table 1:** Characteristics of the study participants and their relationship with Temporal threshold shift (TTS) among iron and steel workers.


DESCRIPTIVE VARIABLE		TEMPORAL THRESHOLD SHIFT, DEFINED AS HEARING THRESHOLD SHIFTS DB >10 DB AT 1000, 2000, 3000 OR 4000 HZ	

		YES	NO

	(N, %)		(N, %)

Personal noise exposure, *LAeq*_,8h_			

85.0–89.0	32 (29.1)	29 (90.6)	3 (9.4)

89.1–92.4	52 (47.3)	45 (86.5)	7 (13.5)

92.5–95.5	26 (23.6)	20 (76.9)	6 (23.1)

All	110 (100.0)	94 (85.5)	16 (14.5)

Age-group (years)			

20–27	41 (37.3)	35 (85.4)	6 (14.6)

28–35	34 (30.9)	31 (91.2)	3 (8.8)

36–47	35 (31.8)	28 (80.0)	7 (20.0)

Duration of work (years)			

0–3.0	41 (37.3)	33 (80.5)	8 (19.5)

3.1–7.0	35 (31.8)	32 (91.4)	3 (8.6)

7.1–20	34 (30.9)	29 (85.3)	5 (14.7)

Current smoking			

No	96 (87.3)	82 (85.4)	14 (14.6)

Yes	14 (12.7)	12 (85.7)	2 (14.3)

Use of earphones/headphones			

No	96 (87.3)	81 (84.4)	15 (15.6)

Yes	14 (12.7)	13 (92.9)	1 (7.1)

	**MEAN (SD)**	**MEAN (SD)**	**MEAN (SD)**

Personal noise exposure, dB(A)	90.4 (2.7)	90.2 (2.6)	91.1 (2.6)
Number of noise peaks >130, dB(C)	12 (9.7)	10 (9)	16 (12)


**Figure 1:**
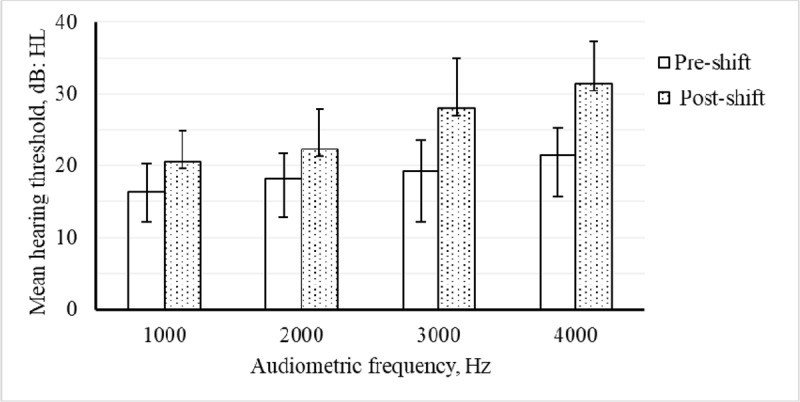
The mean hearing thresholds (dB) at pre- (open boxes) and post- (dotted boxes) shift audiometries with respective standard deviations for 1000, 2000, 3000 and 4000 Hz (N = 55). The difference between the pre- and post-shift hearing thresholds was statistically significant for all frequencies (paired samples t-test, p < 0.001).

In this study, there was a statistically significant increase in the mean hearing threshold shifts from pre- to post-shifts (Paired samples t-tests, two tailed; P < 0.001) at audiometric frequencies of 1000, 2000, 3000, and 4000 (***Figure 1***). The difference in mean hearing thresholds was higher at 4000 Hz [Arithmetic Mean (AM) = 10 dB SD = 4 dB] compared to that of 1000 Hz (AM = 4 dB SD = 3 dB), 2000 Hz (AM = 4 dB SD = 4 dB), and 3000 Hz (AM = 9 dB SD = 6 dB), respectively (***Figure 1***). Also, the largest proportion of hearing threshold shifts >10 dB was recorded at 4000 Hz (about 71%, of the PTA measurements) compared to about 54%, 13% and 18% at 3000, 2000 and 1000 Hz respectively (not shown in the Table).

Overall, 85.5% of workers had a TTS defined as a 10 dB or greater change at 1000, 2000, 3000 or 4000 Hz (***Table 1***). There was no statistically significant association between TTS and personal equivalent noise exposure, number of noise peaks >130 dB (C), age, work duration, current smoking and use of earphones/headphones (***Table 1***). The participant’s age and duration of work were strongly correlated (r = 0.6, P < 0.001). Likewise, the number of noise peaks was significantly correlated to personal noise exposure (Pearson correlation r = 0.5, p < 0.001).

**Figure 2:**
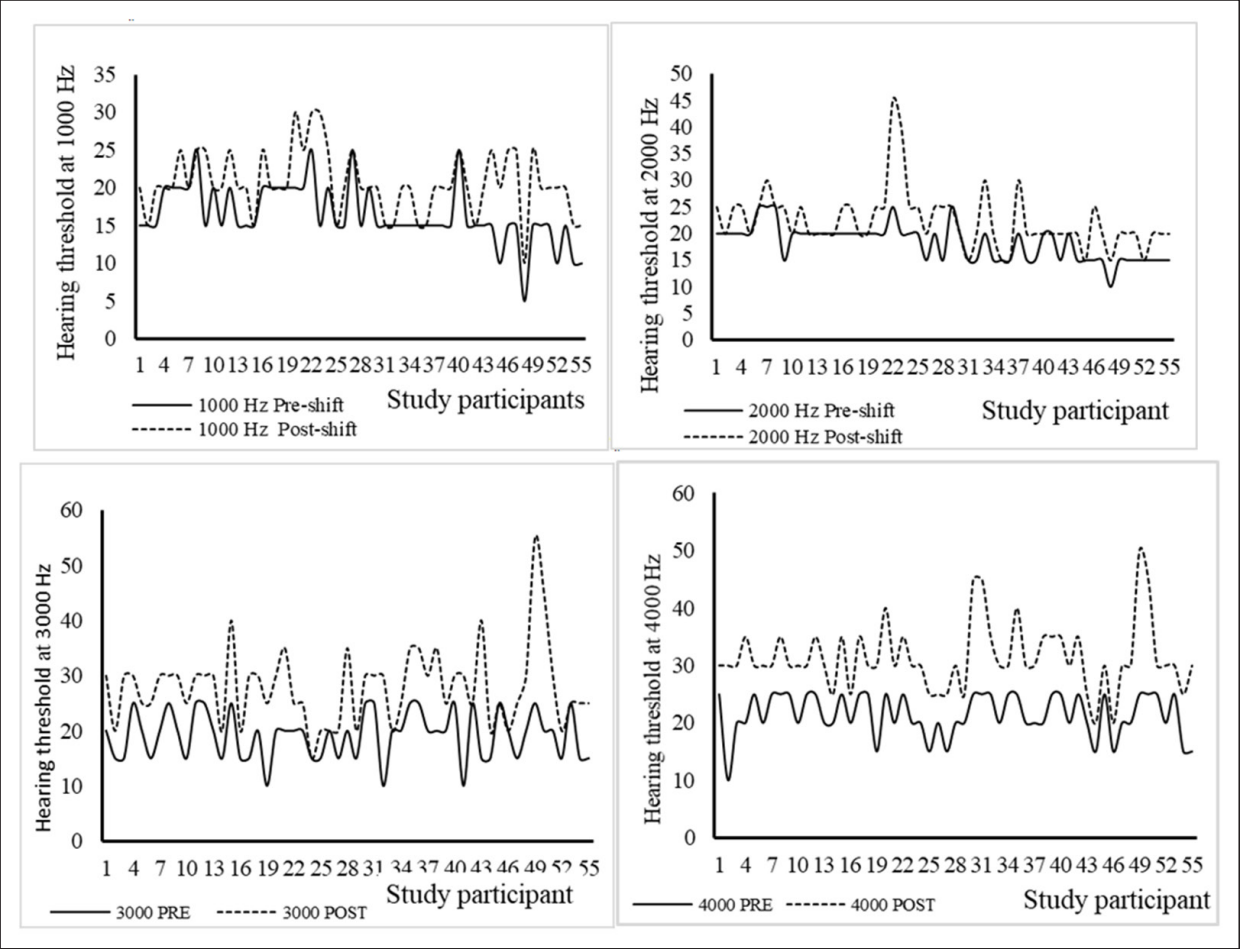
Pre-shift hearing threshold (solid lines) and post-shift hearing threshold (dotted lines) for the frequencies 1000, 2000, 3000 and 4000 Hz among noise-exposed workers (N = 55).

Furthermore, the individual PTA measurements showed that the hearing threshold shifts varied between workers, and generally increased from 1000 Hz to 4000 Hz (***Figure 2***). The largest recorded difference in hearing threshold in individual audiometry were 30 dB at 3000 Hz and 4000 Hz followed by several others of 20 – 25 dBs, many of which were recorded at 4000 Hz (***Figure 2***).

## Discussion

In our study, the prevalence of TTS among the iron and steel workers exposed to a full- shift noise level of 85–95.5 dB(A) was 85.5%. There was a significant increase in mean hearing thresholds across the work shift at all measured noise frequencies. The largest increase in hearing threshold was found at 4000 Hz, suggesting that this audiometric frequency was more sensitive than the lower frequencies to noise exposure. To the best of our understanding, this study is the first to be conducted among iron and steel workers in Tanzania and indicates that registration of TTS might be a useful tool to validate the effect of the planned intervention to reduce occupational noise exposure among workers. If successful, the intervention might reduce the prevalence of TTS, and thereby indicate that the risk of permanent threshold shift (PTS) due to repeated high noise exposures among the workers will be reduced.

Although we could not find any analogous studies from industrial work environments, our study shows a comparable prevalence of TTS as reported among male hockey officials in the U.S i.e., 86.2% [17]. This supports the findings that exposure to high sound levels has an impact on TTS in humans [31,32,33]. Despite the similarities between these two studies regarding the prevalence of TTS, the equivalent and the peak noise exposure (134.1 vs. 134 dBC), the two studies differ in duration of noise exposure since the hockey game officials had a noise exposed period of about three hours, and probably also in sound frequency and temporal patterns [16,34]. On the contrary, studies among bartenders in Poland yielded somewhat lower estimate of TTS (77%) at an average personal noise exposure of 95dB(A) than that of our study. However, the study had relatively few participants, yet results clearly suggest the likelihood of a relationship between the recorded personal noise exposure and the TTS.

In the present study the prevalence of TTS did not increase with an increase in noise exposure. This is in agreement with a study that found no significant correlation between occupational noise exposure of 85 to 90 dB(A) and TTS among iron workers in Norway [35]. These findings are not comparable to the results in Alcoa workers in US [36], which indicated an association between increasing noise exposure and acute hearing loss [6]. However, in this study hearing assessment was performed by subjective self-reports that are likely to be influenced by age, gender, race, education level [37], and/or psychological factors related to the way the question was asked or the question wording itself [31]. One possible explanation of this lack of relationship between increasing noise level and TTS might be attributed to a healthy worker effect. This effect means that healthy workers, with good hearing status, are likely to remain in noisy work, while workers who develop problems with their hearing will likely leave the job or change to a work section with lower noise levels. This is a speculation, and difficult to show in the present study, but this type of selection of workers is well known phenomena in working life [38]. We did not track the workers movement and placement over their previous years of employment and thus it is difficult to make accurate conclusions about this issue and more detailed studies and analyses may be required.

We found a significant increase in hearing threshold across work shift, which was more pronounced at 4000 Hz than at the lower audiometric frequencies. This is analogous with several studies involving human subjects in different sectors, although the magnitude of threshold change varies in different studies. However, what seems to be common to the different studies is the significant role of the noise exposure to observed results and that the higher audiometric frequencies, especially at 4000 Hz, indicates a noise effect on hearing [39,40]. For instance, while we found a mean change in the threshold shift of 10 (SD = 4) dB at 4000 Hz, other studies done among young adult volunteers (aged 18–27 years) in the U.S and Japan (age 18–30 years) exposed to average noise levels of 92–102 dB(A) for 51 minutes and 103.5 – 111.3 dB(A) for four hours, respectively found an increased mean hearing threshold shift of 6.3 (SD = 3.9) dB and 5.2 (SD = 6.9) at 4000 Hz.

There was a large individual variability in cross-shift change in hearing threshold among the iron and steel factory workers which conforms to the already available body of knowledge on the individual variations in susceptibility to noise in various studies [6,19,41]. Different factors have been suggested to explain the variability such as the interactions between noise exposure and other agents such as, ototoxic drugs, carbon monoxide and solvents; non-auditory factors (eye color, age, smoking, diet); and auditory system-related factors (differences in acoustic reflex functioning, the role of the efferent system, and history of noise exposure) [16,27,42].

The strengths of the present study include the use of standard methods and devices in data collection, i.e., the use of calibrated noise dosimeter and audiometer for collecting information in the field while adhering to the ISO standard 9612:2009 and ISO 8253–1:2010 as well. All the audiometric tests were all done in a background noise controlled mobile booth located within the factory during measurement, using similar standard protocol for validity of the results. Potential determinants for TTS were identified and their relationship with TTS was analyzed. In addition, only workers with normal hearing were included. It might have been a strength if we had been able to conduct a follow-up audiometry after 48 hours or more to confirm the presence or absence of TTS and establish a complete recovery to baseline threshold level. However, this was not possible in our setting as workers were obliged to continue working on daily basis afterwards.

The TTS studies are, in some cases, quite complex. One challenge is the availability of standard definitions across studies. Some researchers use the higher noise sensitive frequencies of 3000 – 6000 Hz and others focus on lower audiometry frequencies both as average and or single frequency metrics [17,19,43]. However, in most cases, there is an agreement that the noise sensitive frequencies especially at 4000 Hz should be included. In this study, we have used the definition of a shift of 10 dB or greater change at 1000, 2000, 3000 or 4000 Hz in either ear between two consecutive audiometries [16].

To our knowledge, this is the first study in Tanzania exploring the relationship between occupational noise exposure and TTS among iron and steel workers. However, our results are most likely to be representative for the workers in the same kind of working environments with the same kinds of noise characteristics. Our results seem to agree with findings from similar studies in other parts of the world and seem to be related to the noise levels and not to geography or type of industry. However, it is important to underline that the results are only representative for workers without any protective hearing equipment.

## Conclusion

The findings of our current study were from screened iron and steel workers with normal hearing (with PTA better than 25 dB Hearing Level) who were exposed at an average personal noise exposure of 90.4 dB(A) and above. We found 85.5% of workers had TTS after completing a work shift. Interventions to reduce the occupational personal noise exposure among workers are warranted to reduce the high risk of developing permanent threshold shift with persistent high noise exposure.

## References

[1] Seixas NS, Neitzel R, Stover B, et al. 10-Year prospective study of noise exposure and hearing damage among construction workers. Occupational and Environmental Medicine. 2012; 69: 643–650. DOI: 10.1136/oemed-2011-100578 22693267 PMC4570847

[2] Lie A, Skogstad M, Johannessen HA, et al. Occupational noise exposure and hearing: A systematic review. International Archives of Occupational and Environmental Health. 2016; 89: 351–372. DOI: 10.1007/s00420-015-1083-5 26249711 PMC4786595

[3] Rabinowitz PM. The public health significance of noise-induced hearing loss. In: Le Prell CG, Henderson D, Fay RR, Popper AN, eds. Noise-Induced Hearing Loss: Scientific Advances Springer Handbook of Auditory Research 40. New York, NY, USA: Springer Science + Business Media, LLC. 2012; 13–25. ISBN: 978-1-4419-9523-0_2.

[4] World Health Organization (WHO). Prevention of Blindness and Deafness. Global Estimates on Prevalence of Hearing Loss. 2018. Available online: http://www.who.int/pbd/deafness/estimates/en/. (Accessed September 20, 2020).

[5] Morata TC. Hearing disorders. In: Levy BS, Wegman DH, Baron SL, Sokas RK, eds. Occupational and Environmental Health: Recognizing and preventing disease and injury. 5^th^ ed. Philadelphia, U.S.: Lippincott Williams & Wilkins. 2006; 587–596.

[6] Cantley LF, Galusha D, Slade MD. Early hearing slope as a predictor of subsequent hearing trajectory in a noise-exposed occupational cohort. The Journal of the Acoustical Society of America. 2019; 146: 4044. DOI: 10.1121/1.5132542 31795687 PMC6881190

[7] Yhdego M. Assessment of noise pollution in friendship textile mill limited, Ubungo —Dar es Salaam. Environment International. 1991; 17: 479–485. DOI: 10.1016/0160-4120(91)90282-U

[8] Minja BM, Moshi NH, Riwa P. Noise-induced hearing loss among industrial workers in Dar es salaam. East African Medical Journal. 2003; 80: 238–242. DOI: 10.4314/eamj.v80i6.8705 12953738

[9] John W, Sakwari G, Mamuya SH. Noise exposure and self-reported hearing impairment among gas-fired electric plant workers in Tanzania. Annals of Global Health. 2018; 84: 523–531. DOI: 10.29024/aogh.2305 30835397 PMC6748259

[10] Mdeme FG, Mkoma SL. Assessment of work zone noise levels at a cement factory in Tanga, Tanzania. Ethiopian Journal of Environmental Studies and Management. 2012; 5: 225–231. DOI: 10.4314/ejesm.v5i3.2

[11] Nyarubeli IP, Tungu AM, Bråtveit M, Moen BE. Occupational noise exposure and hearing loss: A study of knowledge, attitude and practice among Tanzanian iron and steel workers. Archives of Environmental & Occupational Health. 2019; 1–10. DOI: 10.1080/19338244.2019.1607816 31033430

[12] Nyarubeli IP, Tungu AM, Moen BE, Bråtveit M. Prevalence of noise-induced hearing loss among Tanzanian iron and steel workers: A cross-sectional study. International Journal of Environmental Research and Public Health. 2019; 16: 1367. DOI: 10.3390/ijerph16081367 PMC6518298 30995750

[13] Nyarubeli IP, Tungu AM, Bratveit M, Sunde E, Kayumba AV, Moen BE. Variability and determinants of occupational noise exposure among iron and steel factory workers in Tanzania. Annals of Work Exposures and Health. 2018; 62: 1109–1122. DOI: 10.1093/annweh/wxy071 30113644 PMC6231025

[14] Prell CG, Henderson D, Fay RR, Popper AN. Prevention of noise-induced hearing loss: Potential theurapituc agents. In: Prell CG, Bao J, eds. Noise-Induced Hearing Loss: Scientific Advances. New York, USA: Springer Science +Business media. 2012; 285–289. DOI: 10.1007/978-1-4419-9523-0_13

[15] Sliwinska-Kowalska M. New trends in the prevention of occupational noise-induced hearing loss. International Journal of Occupational Medicine and Environmental Health. 2020; 33: 841–848. DOI: 10.13075/ijomeh.1896.01600 32994587

[16] Ryan AF, Kujawa SG, Hammill T, Le Prell C, Kil J. Temporary and permanent noise-induced threshold shifts: A review of basic and clinical observations. Otology & neurotology: Official publication of the American Otological Society, American Neurotology Society [and] European Academy of Otology and Neurotology. 2016; 37: e271–e275. DOI: 10.1097/MAO.0000000000001071 PMC4988324 27518135

[17] Adams KL, Brazile WJ. A faceoff with hazardous noise: Noise exposure and hearing threshold shifts of indoor hockey officials. Journal of Occupational and Environmental Hygiene. 2017; 14: 104–112. DOI: 10.1080/15459624.2016.1225158 27540829

[18] Wolniakowska A, Zaborowski K, Dudarewicz A, Pawlaczyk-Luszczynska M, Sliwinska-Kowalska M. Assessment of temporary hearing changes related to work as a bartender. Medycyna Pracy. 2019; 70: 17–25. DOI: 10.13075/mp.5893.00734 30667383

[19] Le Prell CG, Dell S, Hensley B, et al. Digital music exposure reliably induces temporary threshold shift in normal-hearing human subjects. Ear and Hearing. 2012; 33: e44–e58. DOI: 10.1097/AUD.0b013e31825f9d89 22885407 PMC3480981

[20] The United Republic of Tanzania (URT). The Occupational Safety and Health Act of 2003. Accessed September 15, 2020. Available online: http://www.parliament.go.tz/Polis/PAMS/Docs/5-2003.pdf.

[21] Themann CL, Byrne DC, Davis RR, Morata TC, Murphy WJ, Stephenson MR. Early prognosis of noise-induced hearing loss: Prioritising prevention over prediction. Occupational and Environmental Medicine. 2015; 72: 83–84. DOI: 10.1136/oemed-2014-102453 25391832 PMC4589253

[22] National Institute for Occupational Safety and Health (NIOSH). Criteria for A Recommended Standard: Occupational Noise Exposure Revised Criteria 1998. In: NIOSH, ed. Cincinnati, Ohio. Accessed September 1, 2020. Available online: https://www.cdc.gov/niosh/docs/98-126/pdfs/98-126.pdf.

[23] Seixas NS, Neitzel R, Stover B, et al. A multi-component intervention to promote hearing protector use among construction workers. International Journal of Audiology. 2011; 50: S46–S56. DOI: 10.3109/14992027.2010.525754 21091403 PMC4568816

[24] Mills JH, Adkins WY, Gilbert RM. Temporary threshold shifts produced by wideband noise. The Journal of the Acoustical Society of America. 1981; 70: 390–396. DOI: 10.1121/1.386774 7288026

[25] International Standard Organization (ISO). Acoustics – Determination of Occupational Noise Exposure – Engineering method; ISO standard 9612:2009. Geneva, Switzerland; 2009.

[26] International Standard Organization (ISO). Acoustics-Audiometric Test Methods—Part 1: Pure Tone Air and Borne Conduction Audiometry; ISO standard 8253–1: 2010. Geneva, Switzerland; 2010.

[27] Ward WD. Susceptibility to auditory fatigue. Contrib Sens Physiol. 1968; 3: 191–226. 5728652

[28] Ward WD. Diurnal Variability Of Auditory Threshold. Acta Otolaryngol. 1964; 58: 139–142 DOI: 10.3109/00016486409121370 14210223

[29] Franks JR. Hearing measurement. In: Goelzer B, Hansen C, Sehrndt G, eds. Occupational Exposure to Noise: Evaluation, Prevention and Control, World Health Organization. Dortmund: WHO electronic book, Federal Institute for Occupational Safety and Health, 2001; 8: 183–232.

[30] British Society of Audiology (BSA). Recommended Procedure: Pure Tone Air Conduction and Borne Conduction Threshold Audiometry with or without Masking. 80 Brighton Road, Reading Berkshire, RG6 1PS, UK: British Society of Audiology; 2011. Accessed March 12, 2020. Available online: http://www.thebsa.org.uk/wp-content/uploads/2014/04/BSA_RP_PTA_FINAL_24Sept11_MinorAmend06Feb12.pdf.

[31] Brungart DS, Barrett ME, Schurman J, et al. Relationship between subjective reports of temporary threshold shift and the prevalence of hearing problems in military personnel. Trends Hear. 2019; 23. DOI: 10.1177/2331216519872601 PMC6747866 31524086

[32] Melnick W. Human temporary threshold shift (TTS) and damage risk. The Journal of the Acoustical Society of America. 1991; 90: 147–154. DOI: 10.1121/1.401308 1880282

[33] Ward WD. Temporary threshold shift and damage-risk criteria for intermittent noise exposures. The Journal of the Acoustical Society of America. 1970; 48: 561–574. DOI: 10.1121/1.1912172 5470502

[34] Hétu R. Temporary threshold shift and the time pattern of noise exposure. Canadian Acoustics. 1982; 10: 36–44.

[35] Kvaerner KJ. Engdahl B, Arnesen AR, Mair IW. Temporary threshold shift and otoacoustic emissions after industrial noise exposure. Scandinavian Audiology. 1995; 24: 137–141. DOI: 10.3109/01050399509047527 7660058

[36] Rabinowitz PM, Galusha D, Dixon-Ernst C, Slade MD, Cullen MR. Do ambient noise exposure levels predict hearing loss in a modern industrial cohort? Occupational and Environmental Medicine. 2007; 64: 53–59. DOI: 10.1136/oem.2005.025924 16973736 PMC2092595

[37] Kamil RJ, Genther DJ, Lin FR. Factors associated with the accuracy of subjective assessments of hearing impairment. Ear and Hearing. 2015; 36: 164–167. DOI: 10.1097/AUD.0000000000000075 25158982 PMC4272625

[38] Chowdhury R, Shah D, Payal AR. Healthy worker effect phenomenon: Revisited with emphasis on statistical methods – A review. Indian Journal of Occupational and Environmental Medicine. 2017; 21: 2–8. DOI: 10.4103/ijoem.IJOEM_53_16 29391741 PMC5763838

[39] Rabinowitz PM. Noise-induced hearing loss. Am Fam Physician. 2000; 61: 2749–2756, 2759–2760. 10821155

[40] Kirchner DB, Evenson E, Dobie RA, et al. Occupational noise-induced hearing loss: ACOEM Task Force on Occupational Hearing Loss. Journal of Occupational and Environmental medicine/American College of Occupational and Environmental Medicine. 2012; 54: 106–108. DOI: 10.1097/JOM.0b013e318242677d 22183164

[41] Moshammer H, Kundi M, Wallner P, Herbst A, Feuerstein A, Hutter HP. Early prognosis of noise-induced hearing loss. Occupational and Environmental Medicine. 2015; 72: 85–89. DOI: 10.1136/oemed-2014-102200 25063775

[42] Henderson D, Subramaniam M, Boettcher FA. Individual susceptibility to noise-induced hearing loss: An old topic revisited. Ear and Hearing. 1993; 14: 152–168. DOI: 10.1097/00003446-199306000-00002 8344472

[43] Idota N, Horie S, Tsutsui T, Inoue J. Temporary threshold shifts at 1500 and 2000 Hz induced by loud voice signals communicated through earphones in the pinball industry. Annals of Occupational Hygiene. 2010; 54: 842–849. DOI: 10.1093/annhyg/meq048 20584863

